# Are There Sex Differences in Wrist Velocity and Forearm Muscle Activity When Performing Identical Hand-Intensive Work Tasks?

**DOI:** 10.3390/s25175517

**Published:** 2025-09-04

**Authors:** Gunilla Dahlgren, Per Liv, Fredrik Öhberg, Lisbeth Slunga Järvholm, Mikael Forsman, Börje Rehn

**Affiliations:** 1 Department of Epidemiology and Global Health, Umeå University, S-901 87 Umeå, Sweden; gunilla.dahlgren@umu.se (G.D.); lisbeth.slunga-jarvholm@umu.se (L.S.J.); 2 Department of Community Medicine and Rehabilitation, Physiotherapy, Umeå University, S-901 87 Umeå, Sweden; 3 Department of Public Health and Clinical Medicine, Umeå University, S-901 87 Umeå, Sweden; per.liv@umu.se; 4 Section of Biomedical Engineering and Radiation Physics, Department of Diagnostics and Intervention, Umeå University, S-901 87 Umeå, Sweden; fredrik.ohberg@umu.se; 5 IMM Institute of Environmental Medicine, Karolinska Institutet, S-171 77 Stockholm, Sweden; mikael.forsman@ki.se; 6 Division of Ergonomics, School of Engineering Sciences in Chemistry, Biotechnology and Health, KTH Royal Institute of Technology, Hälsovägen 11C, S-141 57 Huddinge, Sweden

**Keywords:** wrist angular velocity, surface electromyography, hand-intensive work, sex difference, risk assessment

## Abstract

Among workers performing hand-intensive tasks, musculoskeletal disorders in the upper extremities are more frequent in women than in men. However, risk assessments are generally not sex-specific, and it is not known whether exposures in regular work differ between females and males. The aim of this study was to compare measured wrist joint velocity and muscle activity between men and women performing identical tasks. Participants (28 female–male pairs) performed one of eighteen hand-intensive on-site tasks. Wrist velocity was measured using inertial units. Forearm muscle activity was measured via surface electromyography and normalized to maximal voluntary electrical activation (MVE). The 10th, 50th, and 90th percentiles and time in muscle recovery (< 0.5 %MVE) were computed. Between-sex differences were tested using the Wilcoxon signed-rank test. Wrist angular velocities did not significantly differ between sexes in any percentile (all *p* > 0.374). The muscle activity was significantly higher in female workers (*p* < 0.001–0.004), ranging from 1.3 to 2.8 times higher, and they spent less time in muscle recovery (*p* < 0.001). In hand-intensive tasks involving women and men, risk assessments should prioritize assessments of women to ensure protection against work-related musculoskeletal disorders for all workers.

## 1. Introduction

Hand-intensive work tasks are performed by approximately one-third of all employees in the USA and EU [1,2] in varied sectors, including industrial assembly, food processing, cleaning, and day nursery work [3]. High repetitivity of hand activity and high hand force are well-known risk exposures for musculoskeletal disorders (MSDs) [4,5,6] and diagnoses such as carpal tunnel syndrome (CTS) [7,8].

MSDs affect both women and men, but women show notably higher prevalences [3,9,10,11,12,13], with double the risk of a diagnosis from the upper extremity or neck compared to men [14]. Diagnosed CTS is approximately 2–4 times more prevalent in women compared to men [15,16,17], and surgically treated CTS is also more common in women than in men [17,18]. Women also exhibit longer sickness absence than men [19]. Our focus is on risk factors for CTS, which are different occupational physical exposures [20]. However, other risk factors that can be influenced by sex include age, obesity, diabetes, pregnancy, wrist fractures, and inflammatory and degenerative joint diseases [21,22,23,24,25,26].

To focus on occupational exposure, MSD sex differences can be explained by segregation, with different exposures related to physical and psychosocial factors in female and male workers. Females and males commonly work in different occupations. When in the same occupation, their job titles are often different, and when the titles are the same, the tasks are different, also reflecting the influence of how work is organized differently between the sexes [27,28,29,30]. A study of Canadian workers found an excess of CTS cases among women even with the same job title [31]. One way to explore further differences would be to concentrate on levels of physical exposure. To promote equality in workers’ health, it is essential to understand whether females and males are exposed differently in identical tasks. Knowledge of wrist velocity and muscle activity exposures in individuals of different sexes performing identical tasks is important for identifying possible risk segregation.

In hand-intensive work, risk assessment is one of the first steps in a systematic work environment management process to protect workers from musculoskeletal disorders. Hand activity and hand force exposure are central measures in hand-intensive work risk assessment [32,33,34,35] and are predictive for CTS [20,36], typically derived from observations. Ideally, these observed exposures would align with direct technical measurements of wrist angular velocity and muscle activity. Observational methods are commonly used in workplaces as they are low-cost and easy to administer, but they have the disadvantage of being influenced by rater bias [37]. Technical methods for measuring movements and muscle activity are primarily used in laboratory settings, but they are increasingly being applied in field studies, particularly in the measurement of posture and movement [38]. Their benefits include the elimination of rater bias, and continuous data provides greater resolution and the possibility of long data collection periods.

Wrist joint angular velocity can be measured with inertial measurement units (IMUs), and muscle activity can be measured with surface electromyography (sEMG). Wrist angular velocity in °/s is an absolute measure of workload. In contrast, sEMG is a relative measure, normalized to the individual’s maximum muscle activity. Males typically have stronger muscles than females, especially in the upper body [39,40], and so, on average, a female worker exhibits higher muscle activity for the same external workload [39,40]. Females and males do not appear to differ in terms of the strength per cross-sectional area of muscle, number of motor units, or ability to activate motor units. Rather, the difference simply arises from men having a greater muscle mass due to testosterone-induced muscle hypertrophy [39]. According to Gallagher and Barbe [41], increased repetition leads to modest increases in MSD risk, but this is force-dependent; that is, when moving the same total load, lifting lighter loads more frequently is better than lifting heavier loads less frequently in order to avoid cumulative damage in musculoskeletal tissue [41,42]. Furthermore, females’ lower strength capacities imply that increased muscle damage would be expected in female muscle tissue. Other factors that can influence MSDs in females and males are age and anthropometry [41].

Action levels have been proposed for several neck and upper extremity MSD risk factors, including wrist angular velocity and muscle activity [43]. For wrist angular velocity, a suggested action level for the 50th percentile over a workday is 20°/s in general, or 15°/s if the work is force-demanding or involves a vibrating tool [43]. The risk for several diagnoses has been found to be more than twice as high in groups with high wrist or upper arm velocity than in those with low velocity. For extensor carpi radialis (ECR) relative muscle activity, action levels are defined in terms of the maximal voluntary electrical activation (MVE). It is suggested that the 50th percentile of normalized sEMG should not exceed 10 %MVE, the 90th percentile should not exceed 30 %MVE, and the muscle recovery time (i.e., the total time with the sEMG level below 0.5 %MVE) should be at least 5% of the workday [43]. Accurate exposure measurements are crucial for understanding MSD and CTS differences between the sexes and making informed decisions to eliminate or reduce risks in workers of both sexes.

To the best of our knowledge, there are no previous studies comparing measured wrist joint angular velocity and forearm muscle activity in female–male pairs of workers performing identical regular hand-intensive work tasks on-site [37]. Previously, wrist velocity has been investigated as a half-day exposure in a rubber manufacturing (RM) and a mechanical assembly (MA) [44] and in house painters [45]. While average wrist velocity did not differ between genders [44,45], women exhibited higher peak velocities [44]. Forearm flexor and extensor muscle activity has been investigated in RM and MA [44], simulated laparoscopic surgery tasks [46], and simulated house painter tasks [47]. The studies showed statistically significantly higher muscle activity in female workers compared to male workers.

Whole-day exposure measures might introduce differences in physical exposure, related to how work is organized, and influence what female and male workers actually do [48]. To focus on physical exposure and to understand if female and male workers are exposed differently, there is a need to investigate wrist angular velocity and muscle activity exposure and to compare female and male workers in pairs undertaking identical regular tasks on-site. The present study aimed to measure wrist angular velocity and relative muscle activity in real-life hand-intensive work tasks and to investigate if there is a difference in objectively measured exposures between female and male workers.

## 2. Materials and Methods

### 2.1. Design and Participants

To answer the research question, a field study was conducted on site at the participants’ workplaces. The participants were workers who executed identical hand-intensive tasks of various types in female–male pairs to enable comparisons. This study is part of a larger project. The data collection for which is described in detail elsewhere [49]. Initially, one of the authors (GD) made telephone contact with fourteen companies in Sweden in sectors involving manual hand-intensive work tasks. Eight companies agreed to participate, covering the following sectors: warehouse work, pharmaceutical production, industrial assembly work, postal service delivery, postal sorting terminal work, postal sorting of direct mail, manual packaging of portion-packed food, and laboratory analysis and pipetting. Reasons for companies to decline participation included major ongoing organizational changes (*n* = 3), only having female workers (*n* = 1), not performing hand-intensive work (*n* = 1), and the COVID-19 pandemic (*n* = 1).

Inclusion criteria for the workers were (a) performing hand-intensive work tasks, and (b) being able to work with their arms without difficulty. The second of these criteria was defined as a score of <1 on a scale (0 = no difficulty; 1 = some difficulty; 2 = a lot of difficulty; 3 = unable to work) that was inspired by, and adapted from, the Work Activity Limitation Scale [50]. Of the 67 workers who fulfilled these criteria and volunteered to participate in the study, 11 were excluded for various reasons: not performing hand-intensive work (*n* = 2), simulated work tasks (*n* = 2), lack of a female–male pair (*n* = 3), and illness (*n* = 4). The final study population consisted of 56 workers, comprising 28 female–male pairs. This study was conducted in accordance with the Declaration of Helsinki and was approved by the Swedish Ethical Review Authority (ref: 2021-01815; date of decision: 20 May 2021). All workers gave informed consent prior to their participation.

### 2.2. Work Tasks

The project recruited participants from several different workplaces involving hand-intensive work who performed many types of manual work tasks with varying degrees of exposure. Our aim was to obtain a broad diversity regarding different intensities of wrist angular velocity and muscle activity to capture a wide range of physical exposure levels.

Workers and employers identified hand-intensive tasks, based on high repetitivity/hand activity and force, with a daily duration of >4 h [32,33,51]. Also, the task should be performed regularly by both a female and a male worker. Only tasks that workers rated greater than 1 in perception of exertion on the Borg CR-10 scale were included. In total, this resulted in 18 hand-intensive work tasks for measurement (Table 1). For some participants, the work environment had features that could be adjusted to the individual worker’s anthropometrics (mostly table heights and chairs); in other cases, there were no adjustable features. Access to working tools did not differ in the same pair. Workers were instructed to perform the tasks they would typically perform in their normal work routines, and there was no encouragement from the researchers to the workers to adjust their work environment. Some participants worked on the same hand-intensive task throughout the entire workday, while others had additional hand-intensive tasks.

### 2.3. Musculoskeletal Complaints and Clinical Examination

Complaints and pain symptoms in the neck, shoulder, arm, and hand were assessed using the Nordic Questionnaire [52], and diagnostic criteria in these areas were assessed by a standardized systematic clinical evaluation, Health Surveillance in Adverse Ergonomic Conditions (HECO) [53,54]. These assessments have been used previously in similar studies [44].

### 2.4. Strength and Anthropometrics

The grip strength of each hand was tested with a Jamar Plus+ Digital Dynamometer (Patterson Medical, China) [49,55]. Body height, body weight, forearm length, and finger abduction [56] were measured as previously described [49].

### 2.5. Technical Measurements

Wrist kinematics and forearm muscle activity was recorded for 15 min during the hand-intensive work task, using previously validated methods [57].

#### 2.5.1. Movement Velocity and Muscle Activity

The DELSYS Trigno Wireless Biofeedback System Duo (Delsys Inc., Natick, MA, USA) was used. Within that system, the triaxial gyroscopes of two IMUs (Trigno Avanti Sensors, 27 × 37 × 13 mm, 14 g, Delsys Inc., Natick, MA, USA) were used to measure the wrist angular velocity [58]. The sampling frequencies of the gyroscopes were 296 Hz for the hand and 222 Hz for the forearm. Each gyroscope had a dynamic range of ± 250°/s. Two sEMG electrodes in the form of silver sensor bars (Trigno Duo Sensors, 25 × 12 × 7 mm, 21 g, 99.9 % Ag, 5 × 1 mm [5 mm^2^], 10 mm center-to-center interelectrode distance, CMMR > 80 dB, signal-to-noise ratio < 0.75 × V, Delsys Inc., Natick, MA, USA) were used to measure the muscle activity of the forearm wrist flexor muscle flexor carpi radialis (FCR) and wrist extensor muscle extensor carpi radialis (ECR) [59].

#### 2.5.2. Location, Preparation, and Attachment of the IMUs and sEMG Sensors

The most active hand during the work task was measured (right: *n* = 55, left: *n* = 1). One IMU was placed on the dorsal hand and midline on the third metacarpal. The other was positioned on the dorsal forearm distally in the midline of the radius and ulna. The IMUs were localized as close to the wrist joint as possible without them coming into contact during maximal wrist extension.

The FCR sEMG electrode pair was located at the forearm, three to four fingerbreadths distal to the midpoint of a line connecting the medial epicondyle and the biceps tendon [60]. The ECR electrode pair was placed on the radial side of the forearm, approximately one-third of the distance of the forearm length from the elbow in a line from the lateral epicondyle [61,62]. The muscle bellies were identified through palpation during voluntary wrist flexion. The surfaces of the electrodes were cleaned with 70% isopropyl alcohol (Medic), air dried, and then dressed with double-sided self-adhesives (Delsys Mini Sensor Adhesive Interface). The skin was shaved (if hairy), sandpapered, and wiped with 70% isopropyl alcohol until slightly reddened. The sensor bars were applied parallel to the muscle fiber direction on the muscle bellies [59]. All sensors and cables were secured with tape (Fixomull stretch), and when necessary, additionally covered with 5 cm and 10 cm self-adhesive wrap (3M Coban NL). Finally, the IMU and sEMG connections were tested.

#### 2.5.3. SEMG Normalization

**Figure 1:**
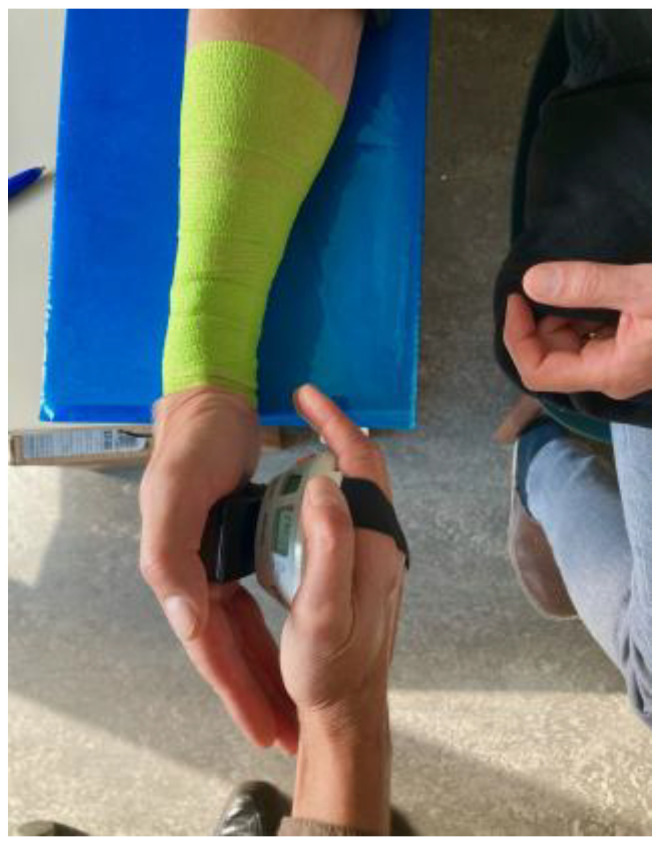
Dynamometer pressure applied for testing MVIC of FCR (palmar).

To enable a comparison of sEMG activity in the same muscles between the female and male workers, the sEMG amplitude was normalized [63]. Prior to the work task, the worker performed three maximal voluntary isometric contractions (MVIC) for 5 s separated by at least 5 s of recovery, for each muscle. The MVIC was tested with the worker comfortably seated on a chair. The forearm was rested on the table at elbow height; it was semi-pronated, with a straight wrist and the hand outside the table’s edge. An anti-slip cloth was placed on the table under the forearm. A dynamometer (MicroFET, Hoggan Scientific, with a round attachment of 4 cm diameter) was used to exert a perpendicular pressure for the worker to resist, centered on the third metacarpal. For FCR, the pressure was applied to the hand, while for ECR, the pressure was applied at the opposing center dorsally of the hand (Figure 1). The worker was verbally encouraged to resist the manually applied force gradually to a maximum. All MVICs were instructed by the same researcher (GD). The highest amplitude of a 0.5 s moving root mean square (RMS) window was considered to be the MVE, which was then used for normalization [63].

#### 2.5.4. Data Processing and Analysis

The IMU and sEMG data were processed using a custom-written MATLAB program (MATLAB R2022b Update 2 [9.13.0.2105380], MathWorks, Natick, MA, USA). The wrist angular velocity for the time in the work task was computed for the 10th, 50th, and 90th percentiles according to the flex method of wrist flexion–extension velocity by Manivasagam and Yang [58]. Only the velocity around the axis of flexion–extension was used.

Before the wrist angular velocity was calculated, the data from the gyroscope were low-pass-filtered using a fourth-order Butterworth filter with a cut-off frequency of 5 Hz, followed by down-sampling to 20 Hz. The wrist velocity was then calculated at each instance in time using the following equation:

$$v=\sqrt{{\left(g_{hand}-g_{forearm}\right)}^{2}}$$

where v represents the velocity, and *g_hand_* and *g_forearm_* represent the angular velocity around the axis of flexion–extension for the hand and forearm, respectively. This way of measuring the velocity with IMUs is known to correlate well with goniometer-measured velocity [58].

The sEMG signals were sampled at 2000 Hz and bandpass-filtered using a fourth-order Butterworth filter with a passband of 20–450 Hz. This was then transformed to an amplitude using an RMS calculation with a 100 ms window in the Trigno Discover software, version 1.5.0 (Delsys). Then, the RMS was normalized to the individual’s MVE; i.e., to a 10 Hz normalized sEMG RMS signal.

### 2.6. Statistical Data Analysis

Statistical calculations were performed using version 28.0.1.1(15) of SPSS Statistics for Windows (IBM, Armonk, NY, USA). Descriptive statistics for continuous variables are presented as mean values and standard deviations if symmetrically (normally) distributed and as medians and quartiles [quartile 1: Q1; quartile 3: Q3] otherwise. Categorical variables are presented as numbers and proportions. The normal distribution of the IMU and sEMG RMS values was checked by inspecting histograms and Q-Q plots [64,65]. Since the IMU and sEMG data were mostly non-normal, the non-parametric Wilcoxon signed rank test was applied uniformly for all variables to assess whether there was a significant difference between the paired females and males. The 10th, 50th, and 90th percentiles of each worker’s wrist angular velocity were computed. For FCR and ECR sEMG muscle activity, each worker’s 10th, 50th, and 90th percentiles were computed; these correspond to measures of static, median, and peak load, respectively [66]. As a measure of muscle recovery, the %time spent with the sEMG amplitude below 0.5 %MVE was calculated [43]. All statistical tests were 2-sided, and the significance level was set at 5%.

## 3. Results

### 3.1. Description of the Workers

Table 2 describes the female and male workers. Males had a higher proportion of workers who reported pain from the neck, shoulder, hand, or forearm. Conversely, females had a higher number of established diagnoses in the neck and upper extremities. All workers were non-smokers (Table 2).

### 3.2. Wrist Angular Velocity—IMUs

There were no statistically significant differences between females and males in terms of wrist angular velocity in any of the three percentiles (Table 3). The median of the workers’ 50th percentile of wrist angular velocity was approximately 20°/s in both sexes.

### 3.3. Muscle Activity—sEMG

Regarding the normalized sEMG RMS muscle activity for the FCR and ECR workload, all three percentiles were significantly higher in females than in males (Table 4). Females also spent significantly less time in muscle recovery (sEMG < 0.5 %MVE). For the FCR, 22 females and 7 males spent less than 5% of the measured time in recovery; the corresponding numbers for the ECR were 24 females and 16 males.

### 3.4. Missing Values

There were no missing values for the IMUs. However, for sEMG, there was a data loss that reduced the number of pairs in the data by 3, leaving 25 pairs (28 pairs minus the 3 missing pairs) for the sEMG measures.

## 4. Discussion

To the best of our knowledge, this is the first study to investigate wrist angular velocity and muscle activity exposures during regular hand-intensive identical work tasks performed by experienced workers in female–male pairs during real conditions. The primary findings were that there were no significant differences in measured wrist angular velocities between the sexes, but measured muscle activity was significantly higher, and time in muscle recovery was significantly shorter in females.

### 4.1. Comparison to Other Studies—Wrist Velocity

As already mentioned, we are not aware of any other studies using female and male workers paired in identical on-site tasks that measure wrist velocity, as in the present investigation. Therefore, the comparison between women and men in identical tasks is extended to measured half-day exposures. The lack of significant sex differences in the 50th percentile (median) in the present study was supported by Heilskov-Hansen et al. [45] in house painters, and by Nordander et al. [44] in rubber manufacturing and mechanical assembly. The present result at the 90th percentile (peak), since there was no significant difference between sexes, conflicted with that of Nordander et al., who found a significantly higher velocity in women. This may be due to differences in measurement duration or task between the studies. The compared studies used electro goniometry, which is similar to IMUs. Taken together, this suggests that wrist velocity in the 50th percentile does not differ between female and male workers in the identical tasks. The lack of sex difference in the 50th percentile may be due to the fact that in tasks performed by female and male workers, wrist velocity reflects productivity speed, e.g., the number of items handled per time unit in this context.

### 4.2. Comparison to Other Studies—Measured Muscle Activity

Due to the limited number of comparable studies of measured forearm FCR and ECR muscle activity (%MVE), the comparison between women and men in identical tasks is extended to measured half-day exposures [44] and simulated work tasks [46,47].

Nordander et al. [44] found significantly higher ECR muscle activity (%MVE) in the 10th and 90th percentiles in female compared to male workers in rubber manufacturing and mechanical assembly, which confirms our results. Meyland et al. [47] also found significantly higher FCR and ECR muscle activity for the 10th, 50th, and 90th percentiles in female compared to male house painters in simulated tasks, which aligns with the results of this study. Kim et al. [46] showed significantly higher FCR and ECR muscle activity (%MVE) in female surgeons compared to male surgeons performing simulated urethroscopy tasks. Females’ muscle activity was higher in the compared positions (sitting/standing) in three different tasks, taking into consideration the types of urethroscopes used. The results of Kim et al. support the present study. In total, all three compared studies showed significantly higher forearm muscle activity in female compared to male workers, aligning with the present results.

Our findings concerning sex differences in muscle recovery time align with those of Thorn et al. [67], who reported significantly higher P10 values in females for trapezius, indicating shorter muscle rest time. The present findings reflect grip strength differences between sexes, with females’ grip strength being approximately 60% that of males, which is consistent with the reference populations [68,69]. When females and males work in identical tasks, females’ relative muscle activity is, as would be expected, higher than that of males due to biological strength differences between the sexes.

### 4.3. Comparison to Recommended Action Levels

For several workers in our study, the measured task was the only task caried out during a workday, or it was executed for ≥4 h per day. Hence, we compared our average values for the female and male groups to suggest whole-day action levels to prevent work-related MSDs in the neck and upper extremities. For wrist velocity, the suggested action limit of 15°/s was exceeded by both the females (median 20°/s) and the males (median 21°/s), meaning that both groups had a high risk of MSDs. Overall, 20 females and 20 males exceeded 15°/s, while 14 females and 15 males exceeded 20°/s.

For ECR muscle activity, only the female workers in this study exceeded the suggested action levels for the median load (12 %MVE vs. the suggested action level of 10 %MVE). Regarding ECR time spent in muscle recovery during the workday, both females (0.0 % time) and males (0.2 % time) failed to reach the suggested muscle recovery time (suggested action level: < 5 % time); hence, both sexes were at risk.

Even though the pairs of workers were performing identical tasks with the same external forces, the proposed muscle activity action levels revealed a higher risk in females than in males.

### 4.4. Strengths

The experienced female and male workers in this study were performing their regular tasks in their real workplace setting, which increases the external validity in comparison to laboratory-based studies. Limiting the data collection to one task enabled a focus on physical exposure and reduced the influence of how work was organized regarding sex. This strengthens the interpretation of the task’s physical exposure and its relation to wrist velocity and muscle activity. Furthermore, one researcher conducted all the measurements to ensure data homogeneity. The sex-paired design, where each pair of workers performed the identical tasks, also strengthens our conclusions.

### 4.5. Methodological Considerations and Limitations

Although industrial sectors were chosen to cover a range of wrist angular velocity and muscle activity exposure levels, our findings may not be generalized to all industries or all forms of hand-intensive work. The workstations could be adjusted to various degrees, e.g., the chair settings, table height, standing or sitting, etc., to fit the worker. Person–workstation/tool fit could have influenced the results comparing female and male workers. In this study, the participants were instructed to work as they normally do, with no advice about altering their behavior or workplace settings given, to preserve real-world working conditions.

The choice of muscles to measure is delicate. We chose the FCR and ECR muscles, which are synergists to the flexor and extensor muscles in the forearm, and which have been used in many previous hand-intensive work studies. When measuring muscle activity, there is often a crosstalk of signals from nearby muscles, partly due to skin movements and partly because the pick-up volume of the surface electrodes may cover several muscles. However, the crosstalk is likely to have been similar between the paired workers, and our interest was not actually in the specific muscles but rather in the individuals’ relative muscle demands in relation to the demanded grip forces.

The sEMG data were normalized by using MVIC in standardized positions for FCR and ECR before the work task, similarly to previous studies [62,70]. Normalization to an MVIC can be affected by difficulties in performing it, which may not accurately reflect the level of muscle activation during dynamic work, but this should also have been similar within each pair. Moreover, the same researcher instructed all participants. There was a limited number of pairs and a limited number of work tasks. For practical reasons, it is rarely possible to include a large number of participants in this kind of study. However, since the sex differences in normalized muscle activity were highly significant, the findings from the current study can likely be generalized to similar settings involving a single task. Further, due to technical issues, we lost sEMG data on three of the worker pairs. This led to a slight loss of statistical power, but the missing measurements were most likely missing completely at random (MCAR) and should therefore not introduce any bias.

### 4.6. Interpretation and Clinical and Theoretical Implications

A credible, sex-inclusive exposure assessment is essential for workplace evaluations. It also forms the basis for targeted actions to prevent work-related disorders. The present results regarding wrist velocity imply that in identical tasks, wrist velocity (°/s) does not, on average, differ when it is measured in a female or a male worker.

In contrast, muscle activity did show a sex-related difference. Females’ significantly higher FCR and ECR muscle activity indicates that to detect sex differences, muscle activity exposure should be assessed. Furthermore, if females and males work in identical tasks, female workers should be prioritized in the assessment of muscle activity exposure to support the protection of all workers.

FCR and ECR sEMG levels were, on average, 1.5 (1.3–2.8) times higher in females than in males, and females also had less muscle recovery time. Together, these findings point to greater continuous muscular load in females. This supports the fatigue failure mechanism theory, which links sustained high muscle activity to an increased risk of musculoskeletal disorders, explaining the higher risk of work-related MSDs in female workers [41]. Furthermore, the proposed action levels [43] imply that due to the relative muscle activity (%MVE), the action levels are sex-independent and can be used to detect muscle activity risk levels for MSDs in females and males equally.

Physical workplace design, such as table height, chair adjustability, or tools, typically designed around male body dimensions, can contribute to sex differences [46]. A better understanding of how workplace design and tools affect wrist velocity and muscle activity in men and women differently would be beneficial; however, this has not been investigated in the present study.

Wrist velocity measures are easier to execute and are also developed for practitioners [58]. SEMG is more typically used in research and rarely used among practitioners in exposure assessment in workplaces [37], and this is perhaps due to the need for more extensive resources such as equipment, knowledge, and time. There is a need for technical devices that are easy to use for practitioners in workplace risk assessment.

### 4.7. Perspectives and Significance

Considering suggested action levels for technical measures, our female participants faced a greater risk of MSDs than their male counterparts. As noted in the introduction to this article, in comparison to men, women exhibit higher prevalences of MSDs and CTS, as well as longer sickness duration. One possible reason for this is that even when work titles are the same, the tasks are often different [27,29,30]. However, in the assembly industry, and among the participants in this study, the sexes often rotate between different workstations in the same work team and in the same way. It therefore looks very likely that female workers’ higher normalized muscle activity contributes to their increased prevalence of MSDs; they are simply exposed to elevated risks and are more prone to injury than their male colleagues. Consequently, it is essential to accurately assess exposure in females involved in hand-intensive work to avoid potential underestimation, especially for muscle activity. Such assessments are crucial for ensuring confidence in risk assessment, inclusive of all workers, for risk-reducing actions, and for ensuring equal protection of all workers. When relying on observational risk assessments, thresholds that adequately protect women become imperative.

## 5. Conclusions

While wrist angular velocity exposure did not seem to differ between females and males performing identical hand-intensive work tasks, differences were found in their normalized muscle activity and muscle recovery exposure, with females showing higher muscle activity and less time spent in muscle recovery than males. These findings have direct implications for risk assessments in hand-intensive work; specifically, it is important that in jobs where women are involved, the risk assessment should be based on females. If observational risk assessment methods are used, the risk criteria level should make it safe for women to perform the job with a low risk of contracting work-related MSDs.

## Figures and Tables

**Table 1:** Hand-intensive work tasks performed by the female–male pairs (*n* = 28).

Work Task	Work Task Description	Number of Pairs
1	manual sorting of mail	4
2	manual sorting of catalogs	2
3	manual sorting of direct mail	2
4	manual packaging of food portions	2
5	ranking of goods	2
6	picking basic food products	2
7	cassette filling	2
8	manual decontamination of bags	2
9	picking fruit/vegetables	1
10	inspection, labeling, and packaging of ampoules	1
11	fluid inspections of bottles	1
12	hose winding	1
13	hose coupling	1
14	picking and scanning small parts	1
15	wheeling	1
16	paternoster picking	1
17	manual pipetting	1
18	water filtration	1

**Table 2:** Description of the workers (*n* = 56) who participated in this study.

	Females	Males
	*n* = 28	*n* = 28
Age ^a^	33 (11.9)	37 (12.2)
Dominant hand, n		
Right	24	28
Left	4	0
Body weight, kg ^b^	66.7 [60.9, 77.5]	92.6 [84.6, 103.8]
Body height, cm ^a^	169.1 (7.7)	182.0 (7.5)
BMI ^b^	24.1 [21.6, 27.4]	27.3 [25.5, 30.9]
Right forearm length, cm [56] ^a^	43.9 (2.1)	48.3 (2.2)
Right finger abduction, cm [56] ^a^	19.8 (1.3)	22.1 (1.5)
Right grip strength, kg [49,55] ^a^	35.5 (6.8)	58.7 (10.0)
Neck/shoulder complaints, last 7 days [41], *n* (%)	18 (46%)	22 (54%)
Hand/forearm complaints, last 7 days [53,54], *n* (%)	9 (32%)	12 (39%)
Neck/shoulder diagnoses [41], *n*		
Tension neck syndrome	1	1
Cervicalgia	1	0
Thoracic outlet syndrome	1	0
Acromioclavicular syndrome right + left	4	1
Biceps tendinitis right + left	4	0
Supraspinatus tendinitis	1	0
Hand/arm diagnoses [41], *n*		
De Quervain, right + left	2	0
Overused hand syndrome, right + left	2	0
Pronator teres syndrome, right + left	0	2
Carpal tunnel syndrome, right + left	0	3
Ulnar nerve entrapment elbow, right + left	0	2
Self-reported work exposures ^b,c^		
Years working with hand-intensive tasks	5.7 [1.5, 13.5]	11.0 [2.3, 22.7]
Hours per day with hand-intensive tasks, repeated movements, and exertion	5.4 (2.0)	5.4 (1.9)
During an intensive day, hours per day with hand-intensive tasks, repeated movements, and exertion	6.0 [5.5, 8.0]	7.0 [6.0, 8.0]

^a^ Mean (standard deviation). ^b^ Median [Q1, Q3]. ^c^ Missing *n* = 1 (female).

**Table 3:** Wrist angular velocity in the 10th, 50th, and 90th percentiles during identical hand-intensive work tasks executed by 28 female–male pairs.

	Wrist Angular Velocity, °/s		
Percentile	Female ^a^	Male ^a^	*p*-value ^b^
10th	2.8 [1.9, 3.6]	2.7 [2.0, 3.5]	0.741
50th	19.9 [14.9, 30.2]	21.1 [13.5, 29.9]	0.374
90th	77.3 [68.6, 108.2]	89.9 [65.7, 108.4]	0.425

^a^ Data are presented as median [Q1, Q3]. ^b^ Wilcoxon signed rank test.

**Table 4:** Muscle activity in terms of maximal voluntary electrical activation (%MVE) in the 10th, 50th, and 90th percentiles during identical hand-intensive work tasks executed by 28 female–male pairs.

	Female ^a^	Male ^a^	*p*-Value ^b^
Flexor carpi radialis			
10th percentile, %MVE	1.4 [1.1, 2.3]	0.5 [0.4, 1.0]	**<0.001**
50th percentile, %MVE	4.3 [3.1, 7.2]	2.2 [1.3, 4.1]	**0.001**
90th percentile, %MVE	13.2 [8.2, 24.5]	9.5 [5.0, 13.7]	**<0.001**
Recovery, % of time ^c^	0.0 [0.0, 1.3]	8.1 [0.2, 19.5]	**<0.001**
Extensor carpi radialis			
10th percentile, %MVE	3.4 [2.6, 5.3]	1.3 [1.0, 2.9]	**<0.001**
50th percentile, %MVE	12.2 [9.8, 15.3]	7.0 [4.7, 9.6]	**0.003**
90th percentile, %MVE	29.3 [24.2, 44.3]	21.8 [16.7, 28.7]	**0.004**
Recovery, % of time ^c^	0.0 [0.0, 0.0]	0.2 [0.0, 4.1]	**0.002**

^a^ Data are presented as median [Q1, Q3]. ^b^ Wilcoxon signed rank test. ^c^ Recovery was defined as time spent at <0.5 %MVE. Bold font indicates statistically significant differences between females and males.

## Data Availability

Individual data on participants used in this study are not publicly available due to personal identifiers in the dataset. Aggregated data is available upon request to gunilla.dahlgren@umu.se.

## References

[1] U.S. Bureau of Labor Statistics Employment by Major Occupational Group. https://www.bls.gov/emp/tables/emp-by-major-occupational-group.htm#top.

[2] Cedefop Employed Population by Sector and Occupation. https://www.cedefop.europa.eu/en/tools/skills-intelligence/employed-population-sector-and-occupation.

[3] Nordander C., Ohlsson K., Akesson I., Arvidsson I., Balogh I., Hansson G.-Å., Strömberg U., Rittner R., Skerfving S. (2013). Exposure–Response Relationships in Work-Related Musculoskeletal Disorders in Elbows and Hands—A Synthesis of Group-Level Data on Exposure and Response Obtained Using Uniform Methods of Data Collection. Appl. Ergon..

[4] Zimbalist A., Rempel D., Feng L., Harris-Adamson C. (2022). The Association Between Forceful Hand Exertions and Musculoskeletal Disorders of the Neck and Shoulder: A Prospective Cohort Study of US Manufacturing Workers. J. Occup. Environ. Med..

[5] Govaerts R., Tassignon B., Ghillebert J., Serrien B., De Bock S., Ampe T., El Makrini I., Vanderborght B., Meeusen R., De Pauw K. (2021). Prevalence and Incidence of Work-Related Musculoskeletal Disorders in Secondary Industries of 21st Century Europe: A Systematic Review and Meta-Analysis. BMC Musculoskelet. Disord..

[6] Balogh I., Arvidsson I., Bjork J., Hansson G.-A., Ohlsson K., Skerfving S., Nordander C. (2019). Work-Related Neck and Upper Limb Disorders—Quantitative Exposure-Response Relationships Adjusted for Personal Characteristics and Psychosocial Conditions. BMC Musculoskelet. Disord..

[7] Hassan A., Beumer A., Kuijer P.P.F.M., van der Molen H.F. (2022). Work-relatedness of Carpal Tunnel Syndrome: Systematic Review Including Meta-analysis and GRADE. Health Sci. Rep..

[8] Gerger H., Macri E.M., Jackson J.A., Elbers R.G., van Rijn R., Søgaard K., Burdorf A., Koes B., Chiarotto A. (2024). Physical and Psychosocial Work-Related Exposures and the Incidence of Carpal Tunnel Syndrome: A Systematic Review of Prospective Studies. Appl. Ergon..

[9] de Kok J., Vroonhof P., Snijders J., Roullis G., Clarke (Panteia) M., Peereboom K., van Dorst P., Isusi I. (2019). Work-Related Musculoskeletal Disorders: Prevalence, Costs and Demographics in the EU.

[10] Andorsen O.F., Ahmed L.A., Emaus N., Klouman E. (2014). High Prevalence of Chronic Musculoskeletal Complaints among Women in a Norwegian General Population: The Tromsø Study. BMC Res. Notes.

[11] Treaster D., Burr D. (2004). Gender Differences in Prevalence of Upper Extremity Musculoskeletal Disorders. Ergonomics.

[12] de Zwart B.C., Frings-Dresen M.H., Kilbom A. (2001). Gender Differences in Upper Extremity Musculoskeletal Complaints in the Working Population. Int. Arch. Occup. Environ. Health.

[13] Wijnhoven H.A.H., de Vet H.C.W., Picavet H.S.J. (2006). Prevalence of Musculoskeletal Disorders Is Systematically Higher in Women than in Men. Clin. J. Pain.

[14] Nordander C., Ohlsson K., Akesson I., Arvidsson I., Balogh I., Hansson G.-A., Strömberg U., Rittner R., Skerfving S. (2009). Risk of Musculoskeletal Disorders among Females and Males in Repetitive/Constrained Work. Ergonomics.

[15] Latinovic R., Gulliford M.C., Hughes R.A.C. (2006). Incidence of Common Compressive Neuropathies in Primary Care. J. Neurol. Neurosurg. Psychiatry.

[16] Tabatabaeifar S., Svendsen S.W., Frost P. (2020). Carpal Tunnel Syndrome as Sentinel for Harmful Hand Activities at Work: A Nationwide Danish Cohort Study. J. Occup. Environ. Med..

[17] Atroshi I., Englund M., Turkiewicz A., Tägil M., Petersson I.F. (2011). Incidence of Physician-Diagnosed Carpal Tunnel Syndrome in the General Population. Arch. Intern. Med..

[18] Linde F., Rydberg M., Zimmerman M. (2022). Surgically Treated Carpal Tunnel Syndrome and Ulnar Nerve Entrapment at the Elbow in Different Occupations and Their Effect on Surgical Outcome. J. Occup. Environ. Med..

[19] Timp S., van Foreest N., Roelen C. (2024). Gender Differences in Long Term Sickness Absence. BMC Public Health.

[20] Yung M., Dale A.M., Kapellusch J., Bao S., Harris-Adamson C., Meyers A.R., Hegmann K.T., Rempel D., Evanoff B.A. (2019). Modeling the Effect of the 2018 Revised ACGIH^®^ Hand Activity Threshold Limit Value^®^ (TLV) at Reducing Risk for Carpal Tunnel Syndrome. J. Occup. Environ. Hyg..

[21] Dahlin L.B., Zimmerman M., Calcagni M., Hundepool C.A., van Alfen N., Chung K.C. (2024). Carpal Tunnel Syndrome. Nat. Rev. Dis. Primers.

[22] Lampainen K., Shiri R., Auvinen J., Karppinen J., Ryhänen J., Hulkkonen S. (2022). Weight-Related and Personal Risk Factors of Carpal Tunnel Syndrome in the Northern Finland Birth Cohort 1966. J. Clin. Med..

[23] Sanjari E., Raeisi Shahraki H., Khachatryan L.G., Mohammadian-Hafshejani A. (2024). Investigating the Association between Diabetes and Carpal Tunnel Syndrome: A Systematic Review and Meta-Analysis Approach. PLoS ONE.

[24] Bland J.D.P. (2023). Carpal Tunnel Syndrome in Pregnancy. Muscle Nerve.

[25] Chung J., Mahmoud Y., Ilyas A.M. (2024). Incidence and Treatment of Carpal Tunnel Syndrome Following Distal Radius Fractures: A TriNetX Analysis of 39,603 Patients. J. Hand Surg. Glob. Online.

[26] Bîrsanu L., Vulpoi G.-A., Cuciureanu D.I., Antal C.D., Popescu I.R., Turliuc D.M. (2024). Carpal Tunnel Syndrome Related to Rheumatic Disease (Review). Exp. Ther. Med..

[27] Biswas A., Harbin S., Irvin E., Johnston H., Begum M., Tiong M., Apedaile D., Koehoorn M., Smith P. (2021). Sex and Gender Differences in Occupational Hazard Exposures: A Scoping Review of the Recent Literature. Curr. Environ. Health Rep..

[28] Quinn M.M., Smith P.M. (2018). Gender, Work, and Health. Ann. Work Expo. Health.

[29] Eng A., Mannetje A., Mclean D., Ellison-Loschmann L., Cheng S., Pearce N. (2011). Gender Differences in Occupational Exposure Patterns. Occup. Environ. Med..

[30] Hagberg M., Forcier L., Kuorinka I. (1995). Work Related Musculoskeletal Disorders (WMSDs): A Reference Book for Prevention.

[31] Kraut A., Rydz E., Walld R., Demers P.A., Peters C.E. (2024). Carpal Tunnel Syndrome among Manitoba Workers: Results from the Manitoba Occupational Disease Surveillance System. Am. J. Ind. Med..

[32] Latko W.A., Armstrong T.J., Foulke J.A., Herrin G.D., Rabourn R.A., Ulin S.S. (1997). Development and Evaluation of an Observational Method for Assessing Repetition in Hand Tasks. Am. Ind. Hyg. Assoc. J..

[33] ACGIH The American Conference of Governmental Industrial Hygienists. https://www.acgih.org/.

[34] Gallagher S., Schall M.C., Sesek R.F., Huangfu R. (2018). An Upper Extremity Risk Assessment Tool Based on Material Fatigue Failure Theory: The Distal Upper Extremity Tool (DUET). Hum. Factors.

[35] Kapellusch J.M., Bao S.S., Malloy E.J., Thiese M.S., Merryweather A.S., Hegmann K.T. (2021). Validation of the Revised Strain Index for Predicting Risk of Incident Carpal Tunnel Syndrome in a Prospective Cohort. Ergonomics.

[36] Violante F.S., Farioli A., Graziosi F., Marinelli F., Curti S., Armstrong T.J., Mattioli S., Bonfiglioli R. (2016). Carpal Tunnel Syndrome and Manual Work: The OCTOPUS Cohort, Results of a Ten-Year Longitudinal Study. Scand. J. Work Environ. Health.

[37] Brambilla C., Lavit Nicora M., Storm F., Reni G., Malosio M., Scano A. (2023). Biomechanical Assessments of the Upper Limb for Determining Fatigue, Strain and Effort from the Laboratory to the Industrial Working Place: A Systematic Review. Bioengineering.

[38] Lind C.M., Abtahi F., Forsman M. (2023). Wearable Motion Capture Devices for the Prevention of Work-Related Musculoskeletal Disorders in Ergonomics-An Overview of Current Applications, Challenges, and Future Opportunities. Sensors.

[39] Haizlip K.M., Harrison B.C., Leinwand L.A. (2015). Sex-Based Differences in Skeletal Muscle Kinetics and Fiber-Type Composition. Physiology.

[40] Miller A.E., MacDougall J.D., Tarnopolsky M.A., Sale D.G. (1993). Gender Differences in Strength and Muscle Fiber Characteristics. Eur. J. Appl. Physiol. Occup. Physiol..

[41] Gallagher S., Barbe M.F. (2022). Musculoskeletal Disorders: The Fatigue Failure Mechanism.

[42] Gallagher S., Heberger J.R. (2013). Examining the Interaction of Force and Repetition on Musculoskeletal Disorder Risk: A Systematic Literature Review. Hum. Factors.

[43] Arvidsson I., Dahlqvist C., Enquist H., Nordander C. (2021). Action Levels for the Prevention of Work-Related Musculoskeletal Disorders in the Neck and Upper Extremities: A Proposal. Ann. Work Expo. Health.

[44] Nordander C., Ohlsson K., Balogh I., Hansson G.-Å., Axmon A., Persson R., Skerfving S. (2008). Gender Differences in Workers with Identical Repetitive Industrial Tasks: Exposure and Musculoskeletal Disorders. Int. Arch. Occup. Environ. Health.

[45] Heilskov-Hansen T., Svendsen S.W., Thomsen J.F., Mikkelsen S., Hansson G.-Å. (2014). Sex Differences in Task Distribution and Task Exposures among Danish House Painters: An Observational Study Combining Questionnaire Data with Biomechanical Measurements. PLoS ONE.

[46] Kim E., Sun A., Rodriguez-Alvarez J.S., Ho L., O’Laughlin K., De S. (2024). Gender Differences in Ergonomics during Simulated Ureteroscopy. Am. J. Surg..

[47] Meyland J., Heilskov-Hansen T., Alkjær T., Koblauch H., Mikkelsen S., Svendsen S.W., Thomsen J.F., Hansson G.-Å., Simonsen E.B. (2014). Sex Differences in Muscular Load among House Painters Performing Identical Work Tasks. Eur. J. Appl. Physiol..

[48] Migliore M.C., Ricceri F., Lazzarato F., d’Errico A. (2021). Impact of Different Work Organizational Models on Gender Differences in Exposure to Psychosocial and Ergonomic Hazards at Work and in Mental and Physical Health. Int. Arch. Occup. Environ. Health.

[49] Dahlgren G., Liv P., Öhberg F., Slunga Järvholm L., Forsman M., Rehn B. (2022). Ratings of Hand Activity and Force Levels among Women and Men Who Perform Identical Hand-Intensive Work Tasks. Int. J. Environ. Res. Public Health.

[50] Gignac M.A.M., Badley E.M., Lacaille D., Cott C.C., Adam P., Anis A.H. (2004). Managing Arthritis and Employment: Making Arthritis-related Work Changes as a Means of Adaptation. Arthritis Rheum..

[51] Borg G.A. (1982). Psychophysical Bases of Perceived Exertion. Med. Sci. Sports Exerc..

[52] Kuorinka I., Jonsson B., Kilbom A., Vinterberg H., Biering-Sørensen F., Andersson G., Jørgensen K. (1987). Standardised Nordic Questionnaires for the Analysis of Musculoskeletal Symptoms. Appl. Ergon..

[53] Ohlsson K., Attewell R.G., Jonsson B., Ahlm A., Skerfving S. (1994). An Assessment of Neck and Upper Extremity Disorders by Questionnaire and Clinical Examination. Ergonomics.

[54] Jonker D., Gustafsson E., Rolander B., Arvidsson I., Nordander C. (2015). Health Surveillance under Adverse Ergonomics Conditions—Validity of a Screening Method Adapted for the Occupational Health Service. Ergonomics.

[55] Fess E., Moran C. (1981). Clinical Assessment Recommendations.

[56] PeopleSize Visual Anthropometry Software. https://www.openerg.com/psz/index.html.

[57] Hubaut R., Guichard R., Greenfield J., Blandeau M. (2022). Validation of an Embedded Motion-Capture and EMG Setup for the Analysis of Musculoskeletal Disorder Risks during Manhole Cover Handling. Sensors.

[58] Manivasagam K., Yang L. (2022). Evaluation of a New Simplified Inertial Sensor Method against Electrogoniometer for Measuring Wrist Motion in Occupational Studies. Sensors.

[59] Trigno Wireless Biofeedback System User’s Guide. https://www.delsys.com/downloads/USERSGUIDE/trigno/wireless-biofeedback-system.pdf.

[60] Perotto A.O. (2011). Anatomical Guide for the Electromyographer; The Limbs and Trunk.

[61] Nordander C. (2004). Precision of Measurements of Physical Workload during Standardised Manual Handling. Part I: Surface Electromyography of m. Trapezius, m. Infraspinatus and the Forearm Extensors. J. Electromyogr. Kinesiol..

[62] Dahlqvist C., Enquist H., Löfqvist L., Nordander C. (2020). The Effect of Two Types of Maximal Voluntary Contraction and Two Electrode Positions in Field Recordings of Forearm Extensor Muscle Activity during Hotel Room Cleaning. Int. J. Occup. Saf. Ergon..

[63] Besomi M., Hodges P.W., Clancy E.A., Van Dieën J., Hug F., Lowery M., Merletti R., Søgaard K., Wrigley T., Besier T. (2020). Consensus for Experimental Design in Electromyography (CEDE) Project: Amplitude Normalization Matrix. J. Electromyogr. Kinesiol..

[64] Kim H.-Y. (2012). Statistical Notes for Clinical Researchers: Assessing Normal Distribution (1). Restor. Dent. Endod..

[65] Kim H.-Y. (2013). Statistical Notes for Clinical Researchers: Assessing Normal Distribution (2) Using Skewness and Kurtosis. Restor. Dent. Endod..

[66] Jonsson B. (1982). Measurement and Evaluation of Local Muscular Strain in the Shoulder during Constrained Work. J. Hum. Ergol..

[67] Thorn S., Søgaard K., Kallenberg L.a.C., Sandsjö L., Sjøgaard G., Hermens H.J., Kadefors R., Forsman M. (2007). Trapezius Muscle Rest Time during Standardised Computer Work—A Comparison of Female Computer Users with and without Self-Reported Neck/Shoulder Complaints. J. Electromyogr. Kinesiol..

[68] Mathiowetz V., Kashman N., Volland G., Weber K., Dowe M., Rogers S. (1985). Grip and Pinch Strength: Normative Data for Adults. Arch. Phys. Med. Rehabil..

[69] Massy-Westropp N.M., Gill T.K., Taylor A.W., Bohannon R.W., Hill C.L. (2011). Hand Grip Strength: Age and Gender Stratified Normative Data in a Population-Based Study. BMC Res. Notes.

[70] Ngo B.P.T., Wells R.P. (2016). Evaluating Protocols for Normalizing Forearm Electromyograms during Power Grip. J. Electromyogr. Kinesiol..

